# Application of Pattern Recognition and Computer Vision Tools to Improve the Morphological Analysis of Microplastic Items in Biological Samples

**DOI:** 10.3390/toxics11090779

**Published:** 2023-09-13

**Authors:** Aleksander Maria Astel, Paulina Piskuła

**Affiliations:** Environmental Chemistry Research Unit, Institute of Geography, Pomeranian University in Słupsk, 22a Arciszewskiego Str., 76-200 Słupsk, Poland; paulina.piskula@upsl.edu.pl

**Keywords:** microplastics, binary image, OpenCV, Union–Find algorithm, contour tracking, shape descriptors

## Abstract

Since, in many routine analytical laboratories, a stereomicroscope coupled with a digital camera is not equipped with advanced software enabling automatic detection of features of observed objects, in the present study, a procedure of feature detection using open-source software was proposed and validated. Within the framework of applying microscopic expertise coupled with image analysis, a set of digital images of microplastic (MP) items identified in organs of fish was used to determine shape descriptors (such as length, width, item area, etc.). The edge points required to compute shape characteristics were set manually in digital images acquired by the camera coupled with a binocular, and respective values were computed via the use of built-in MotiConnect software. As an alternative, a new approach consisting of digital image thresholding, binarization, the use of connected-component labeling, and the computation of shape descriptors on a pixel level via using the functions available in an OpenCV library or self-written in C++ was proposed. Overall, 74.4% of the images were suitable for thresholding without any additional pretreatment. A significant correlation was obtained between the shape descriptors computed by the software and computed using the proposed approach. The range of correlation coefficients at a very high level of significance, according to the pair of correlated measures, was higher than 0.69. The length of fibers can be satisfactorily approximated using a value of half the length of the outer perimeter (r higher than 0.75). Compactness and circularity significantly differ for particles and fibers.

## 1. Introduction

In recent years, small pieces of plastic called microplastics have attracted public attention. Due to the misuse and segregation of plastic waste, plastic is released into the environment, and after fragmentation, it spreads across compartments and living organisms. Most plastic wastes can persist in the environment for hundreds of years [1]. MPs are usually considered to be contaminants of synthetic origin with a size of less than 5 mm [2]. However, according to the latest reports, plastic items are classified as nanoplastic (<1 μm), microplastic (1 μm–5 mm), and macroplastic (>5 mm) [3]. Plastic items can be of primary or secondary origin. Primary MPs are directly released from industrial production, while secondary MPs are larger plastic items that break down into smaller pieces, mainly due to the weathering effect [4].

Microplastics have been reported in freshwater [5,6], groundwater [7,8], soil [9,10], sediment [11,12], snow [13,14], ice [15,16], and air [17,18]. Many studies have shown that microplastics are ingested by various organisms [19,20,21], invertebrates [22,23], mammals [24,25], and birds [26,27].

An increasing quantity of MPs present in the environment makes the control of microplastic pollution crucial for environmental and animal protection. Once ingested by aquatic organisms, MPs can trigger physical and toxic effects [28]. A false feeling of fullness [29], damage or blockage of the digestive tract [30], suffocation, and starvation [31] comprise a few of the physical effects. The toxic effect of MPs on aquatic organisms is mainly due to chemicals added to the plastic during manufacturing or absorbed from the environment [32]. The high surface area to volume ratio, as well as the hydrophobic nature of MPs, support the sorption of xenobiotics [33]. Consequently, MPs can cause oxidative stress, neurological damage, endocrine disruption, and immune injury [34].

Large incoherency concerning the identification and quantification of MPs leads to difficulties in comparing MP abundance through various research studies. Generally, an analysis of MPs consists of two phases, namely, the physical characterization of items, followed by chemical characterization, which confirms the chemical nature of the items found. To fulfill the first phase, some initial steps such as extraction from environmental or biological samples, isolation, identification, and rough characterization have to be carried out [35,36]. All the above-mentioned steps are a potential source of analytical uncertainty. For example, to adequately quantify MPs, restrictive procedures to avoid airborne contamination have to be secured [37,38]. Various approaches are commonly used to fulfill the second phase concerning the detection and quantification of MPs in environmental and biological matrices: microscopy [39,40], pyrolysis combined with gas chromatography [41,42], Fourier-transform infrared (FTIR) spectroscopy [43,44], micro-FTIR [5,45], and scanning electron microscopy [41,46]. Both phases require the highest levels of experience since methodological issues challenge data produced by researchers. Usually, inexperienced students or researchers overestimate the abundance of MP items compared to expert researchers [47].

While MPs research has advanced, standard operation protocol concerning the preparation and analysis of samples for the identification of MPs is still not available [48]. Due to the variety of different protocols used and the generation of results of doubtful quality, the comparison of data concerning MP abundance, morphometric features, coloration, and chemical composition is highly ambiguous. Even if contamination prevention is satisfactory, substantial doubts could be related to methodologies of MP size estimation based on digital images acquired through microscopic analysis. The microscopic identification of MPs with dimensions of 0.1–1 mm is one of the easiest and least expensive methods of analysis; however, a false positive or negative interpretation may occur [48]. Moreover, the isolated particles are often incoherently classified since only some microscopes have a camera and software that allows for a detailed approximation of MPs’ shape descriptors. Rarely, MPs are classified according to their shapes (i.e., fibers, fragments, films, granules, foams) and color (mainly transparent, white, red, blue, black, brown, and green), while their largest cross-section is sometimes estimated using calipers [49] or sieve meshes of 2.0, 1.0, 0.5, and 0.25 mm [50]. More often, MPs are classified according to their shapes, color, and arbitrarily selected sets of shape descriptors (morphological features). In addition to shape class and color, morphological features that are usually defined consist of size [51,52,53], size ranges [54,55,56], maximum or undefined length [57,58,59], length and width [60], longest and shortest axes [61], length and diameter [62], longest dimension [63,64,65,66,67,68,69,70,71], length and thickness [72], and the length of fibers and cross-section of other particles [73]. Unfortunately, according to size and other shape descriptors, usually, authors do not specify if the size was measured via the use of functionalities of the built-in software coupled with the microscope, software dedicated to pattern recognition, digital image analysis, or by any other software that allows for working with images. Moreover, very often, it is unknown if morphological features are measured manually (i.e., by putting a dimension line along an estimated height of an item) or automatically (i.e., by creating a bounding box around a measured item). Additionally, as summarized above, a variety of terms and definitions of shape descriptors can be used.

Since many questions concerning the methodology of shape descriptors determination have arisen, the main objective of this study was to propose a procedure based on an application of pattern recognition and computer vision tools that can significantly improve the morphological analysis of microplastic items found in various environmental matrices (including biological ones) via the analysis of digital images taken by using any digital camera coupled with a microscope, even without any dedicated software.

## 2. Materials and Methods

### 2.1. Fish Samples

Five fish species were analyzed: Baltic herring (*Clupea harengus*), Baltic cod (*Gadus morhua*), flounder (*Platichthys flesus*), long-spinded bullhead (*Taurulus Bubalis*), and lumpfish (*Cyclopterus lumpus*). Microplastic items were sought in the gills, digestive tract, and liver. Specimen collection details, the steps of microplastic extraction, and the contamination prevention procedure used can be found elsewhere [74].

### 2.2. Microplastic Identification via the Use of Microscopic Analysis

**Figure 1:**
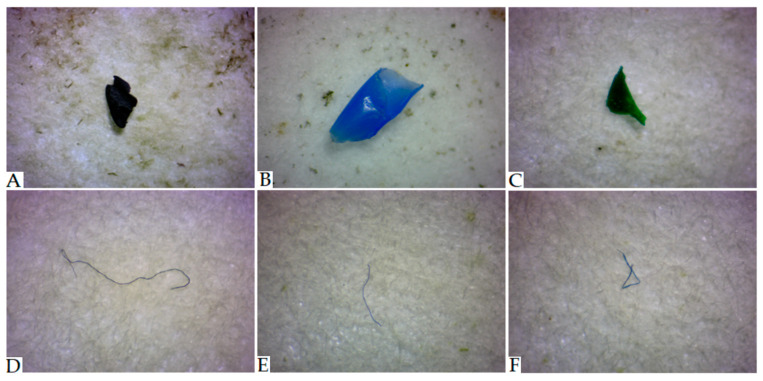
Images of MPs, which were acquired using the Motic Zoom SMZ-161-BLED stereomicroscope coupled with a digital camera ((**A**–**C**)—particles; (**D**–**F**)—fibers).

A Motic Zoom SMZ-161-BLED (Motic, Cabrera de Mar, Spain) stereoscope binocular was employed. It was equipped with 3W LED illumination and the Greenough optical system and connected to a tablet Moticam BTW8 (Xiamen, China) running under the control of the Android 5.0 operating system. Additionally, 24-bit RGB (2048 × 1536 square pixels) images were acquired using 0.75×–4.5× magnification. The edge points used to calculate shape descriptors through the use of built-in software were set manually. The morphometric features of the MP items were measured and archived using MotiConnect 1.5.9.10-build-171215 software, which is a dedicated image processing Android app for Motic cameras. It includes image preview, capture, recording, editing, and basic measuring functions. Some examples of images of MP items acquired by a stereomicroscope coupled with a digital camera are presented in Figure 1. Suspected plastic items were assessed following the protocols recommended by Hidalgo-Ruz et al. [75], Crawford and Quinn [76], and Zobkov and Esiukova [77]. Items with no visible tissue or cell structure of relatively uniform color distribution along the particle and fibers with homogenous diameters along their length were counted as MPs. The other objects were counted as minerals. An analogical procedure for the microscopic determination of MPs was applied by Wang et al. [63].

### 2.3. Software and Hardware

Microsoft Visual Studio Community 2022 ver. 17.1.4 was used to build a project written in C++ with the OpenCV library included. The OpenCV computer vision library [78,79] was chosen as the most popular open-source library that contains a variety of useful algorithms to analyze features in digital images. The visual Studio programming environment was coupled with a default compiler and controlled by the 64-bit Windows 10 home operating system running on an Intel^®^ Core™ i5-8250U CPU 1.6 GHz personal computer with 16.0 GB RAM.

Statistical testing was carried out using the non-parametric U Mann–Whitney test for two-group comparison (particles vs. fibers) since an assumption concerning normal data distribution (tested for circularity and compactness) was not fulfilled. Correlations were assessed using the Pearson correlation coefficient value. All tests were analyzed and considered significant at a *p*-value < 0.001. Statistical analyses were carried out using TIBCO Statistica 13.3 software (TIBCO, Palo Alto, CA, USA).

### 2.4. Image Pretreatment

In total, 125 images were acquired and analyzed. A subset of them was further analyzed according to the procedure described below. It is worth emphasizing that the expectation of better results was not based on the better resolution of the input images, which were of the same resolution as those treated manually; instead, our expectation was based on pixel-level accuracy. Moreover, the use of deep learning algorithms was not intended at the current stage since, in our opinion, a rough number of a hundred images is not sufficient to make learning accurate. Despite this decision, the use of deep learning algorithms in the analysis of digital images of MPs seems to be an interesting and promising direction for future research. 

The purpose of the digital image pretreatment was to obtain, from its initial RGB form, a binary image containing the item silhouette. Although an RGB image could be useful for feature analysis, such as MP color, it was discarded since the determination of the morphological shape descriptors was prioritized. In specialized automatic pattern recognition and computer vision software, some initial pretreatment steps, such as segmentation, border killing, hole filling, and debris removal, are usually triggered before the analysis of morphometric features; however, we decided to minimize the number of pretreatment steps since no item individualization was required because an image of a single item present in an observation field was acquired in each case. Unfortunately, discarding several additional advanced pretreatment steps, the proposed method in its actual form cannot be generalized to different image conditions without compromising performance, however it can be applied directly to images presenting items of the color at least slightly different in comparison with the color of the filter.

**Figure 2:**
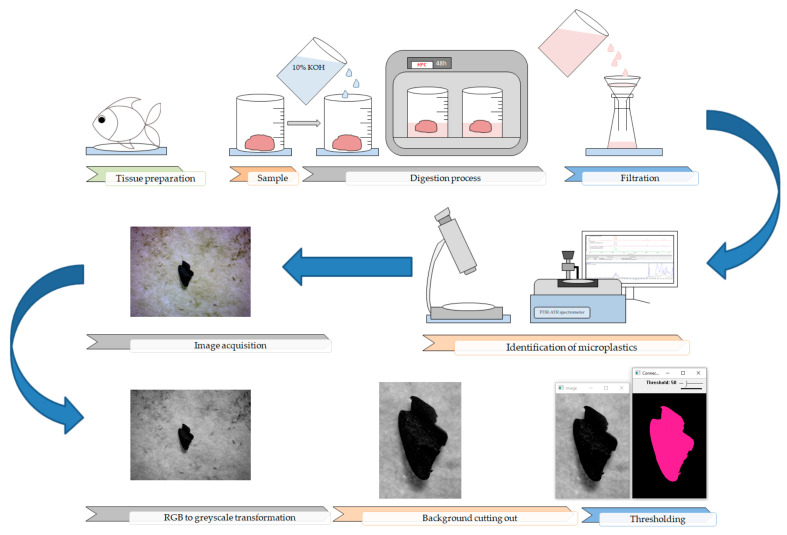
Schematic diagram of microplastic extraction, identification protocol, and digital image pretreatment steps.

Figure 2 presents a schematic diagram of the MP extraction and identification protocol used, along with the pretreatment steps executed for the digital images of MP items, characterized by a color that is darker or lighter in comparison with the color of the filter. The applied steps consisted of (i) RGB to grayscale transformation, (ii) cutting out of a redundant background, (iii) thresholding based on analysis of the distribution of the pixel’s intensities.

The cutting out of a redundant background step was carried out to minimize the size of the images that were to be analyzed by the algorithms and hence minimize computational time; however, the performance improvement concerning the computational time of the C++ app itself was not a priority in this study. It could be further achieved by creating a data store container using histogram-derived thresholding based on the Otsu method, using loops, efficiently allocating memory, etc.; however, every improvement in the code should be further benchmarked. Thresholding produced a binary image, where all pixels with intensities above (or below) a given threshold value are denoted as 1, while all other pixels are denoted as 0. 

**Figure 3:**
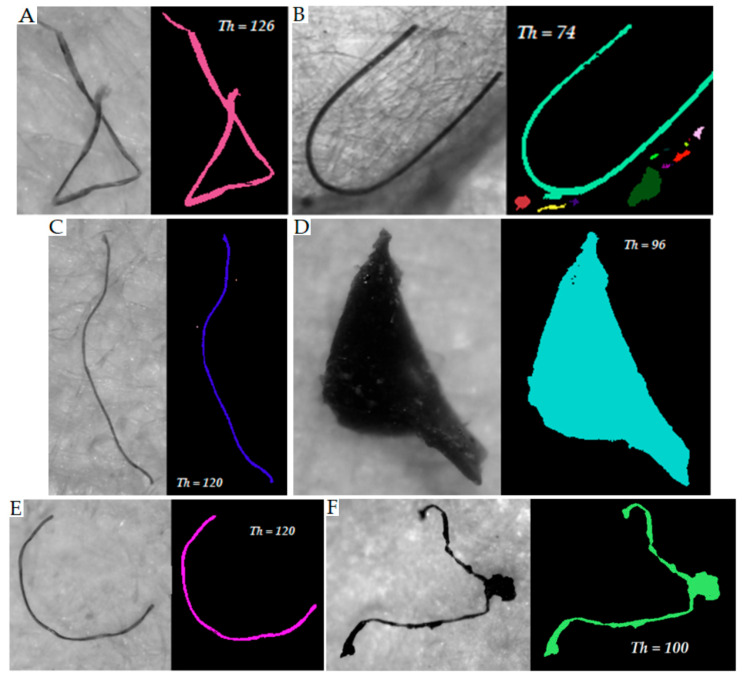
Greyscale images and thresholded images ((**A**–**C**,**E**)—fibers; (**D**,**F**)–particles). Th—threshold value.

**Figure 4:**
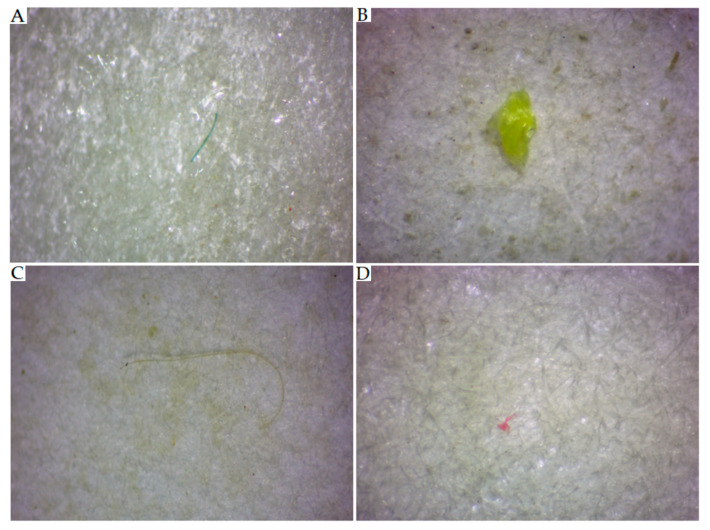
RGB images of MPs that were characterized by the similarity between their color and that of the background ((**A**,**C**)—fibers; (**B**,**D**)—particles).

According to the specific color of the MP items, a threshold value in the range between 52 and 127 was used for items darker than the background. Some examples of greyscale images and MP item shapes obtained via thresholding are presented in Figure 3, while examples of images of transparent MPs that were unsuitable for treatment via background thresholding are shown in Figure 4. 

### 2.5. Union–Find Algorithm

**Figure 5:**
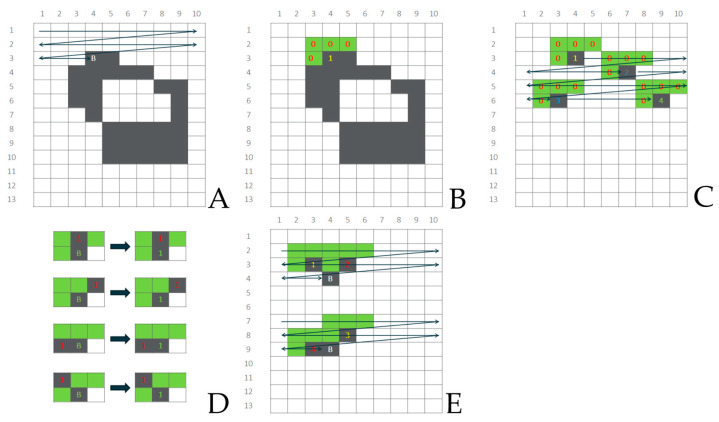
Schematic diagram of applying a label to pixel B according to the eight-connected mask used in the Union–Find algorithm: (**A**) row-wise scanning; (**B**) initiation of the region labeling triggered by the pixel that fulfills the starting criteria; (**C**) cases when a new label is applied; (**D**) cases when already used label is applied to the pixel; (**E**) cases when two separate labels belong to the same region.

Once the pretreatment steps were carried out, the detection of regions was performed using the Union–Find algorithm [80], which is one of the current state-of-the-art algorithms used in so-called connected-component labeling (CCL) tasks. Although there are many other state-of-the-art algorithms used in CCL for which detailed pros and cons discussion can be found elsewhere [81], the Union–Find algorithm was selected since it is available via an OpenCV and easy to self-code. The Union–Find algorithm requires a binary image as an input, with ones and zeros denoting the region (object) and background pixels, respectively. In its basic version, it completes the detection and labeling of regions by forward scanning a given image and using the Union–Find–tree strategy [81]. The detailed theoretical background of the Union–Find algorithm, as well as a variety of references concerning its performance optimization, can be found elsewhere [82], and this is why only a general idea of it is presented in Figure 5 and briefly described below. In the binary image, scanning begins from the top-left pixel coordinates and continues downwards for each pixel from left to right (Figure 5A). When the first pixel of the region is found, a new label is given to it (Figure 5B), and its right-located neighbor is consecutively examined. When a right-located pixel also belongs to a region, the same label is used (Figure 5D). To detect if a new label (Figure 5C) or a label already used should be applied (Figure 5D,E), a mask for the eight-connected connectivity [83] of the currently examined pixel is tested. In the last case, when criteria to initiate a new label for the same region are met several times (Figure 5E), an algorithm finds a union of labels and applies a minority of them to the currently examined pixel, simultaneously creating and storing a tree of dependencies between unified labels. Once image scanning and labeling are complete, an input image with various labels applied to the pixels of regions is scanned again, and a unique label value per region is set according to a tree of dependencies between already unified labels.

### 2.6. Basic Descriptors

Once regions were labeled, they were sorted in descending order according to the sum of pixels (area) belonging to each region. Consecutively, a single region with the highest number of pixels was selected, and then a set of morphometric (shape) descriptors was computed using functions available in an OpenCV library. When an appropriate function was not included in a library, a self-written C++ code was used instead. A set of shape descriptors consisting of an area, outer perimeter length, circularity, minimal and maximal ferret, smallest rectangle, elongatedness, ellipse axes, and compactness were computed using the CCL-based approach. Using the resolution of the image, information concerning the magnification used and the linear dimensions of single-pixel-based shape descriptors were recalculated to obtain values in SI units. Since the computation of an area based on the smallest rectangle and ellipse characteristics seems to be senseless for fibers, in this case, only pixel-based area was calculated.

#### 2.6.1. Area

The area is defined as the number of pixels of a region. Through knowing the size of an image in pixels, it is possible to compute both the width and height of the pixel and, as a result, its area (expressed in the International System of Units (SI)). The advantage of calculating area using pixel coordinates is that it is much more precise and less prone to overestimation as in the case of area computed using the multiplication of the width and length of a given object measured manually.

#### 2.6.2. Perimeter

The perimeter is defined as the total length of the contour of the region, including the outer contour and single or multiple inner contours if the object has a hole or many holes, respectively. In the case of MP particles and pellets, usually, only an outer contour needs to be computed, while in the case of fibers, both outer and inner contours have to be computed since fibers can become twisted (as presented in Figure 2B). In the latter case, through using the perimeter length, a fiber length can be estimated as well. To compute the perimeter length of a given region, a contour tracing algorithm was primarily used [84]. This type of algorithm computes a set of contour points divided into outer and inner contours, respectively, using some hierarchy of parent and child contours. Finally, through using the coordinates of all contour points, the perimeter length is calculated. The distance between two neighboring contour points parallel to the coordinate axes is rated as 1, while the distance in the diagonal is rated as a square root of 2. The perimeter can also be computed using some modified equations, taking into consideration corner issues [85]; however, sub-pixel accuracy was not a priority in this case.

#### 2.6.3. Circularity

Circularity is the measure of how closely the shape of the region approaches the shape of the circle. It can be computed using an area of the region expressed in pixels (*F*) and the maximum distance from the center of gravity of the region to all contour pixels (outer and inner) (*d*), according to the following equation:

$$Circularity=\frac{F}{d_{\max}^{2}\pi }$$

In general, the circularity of the perfect mathematical circle is 1, while if the region is somehow elongated or has holes, the circularity is smaller than 1.

#### 2.6.4. Ferret Diameters

The ferret of a region is defined as the distance between two parallel lines restricting the object perpendicular to that direction. To determine the minimal and maximal ferret, the convex hull of the region is identified, and the so-called rotating calipers algorithm is used to compute the ferret diameters. An algorithm enumerates the antipodal pairs of the convex polygon; that is, it enumerates pairs of points that can be touched at the same time by the two caliper arms. The minimal diameter is the narrowest caliper measure possible for the region. On the contrary, the maximal diameter is the widest caliper measure possible for the region. Using a square as the example, the minimal ferret is the distance between two perpendicular edges of the square, while the maximal ferret is the distance between two square corners located along the diagonal.

#### 2.6.5. Smallest Rectangle

The smallest rectangle of the region is defined as the rectangle with any orientation with the smallest area of all rectangles containing the region.

#### 2.6.6. Elongatedness

Elongatedness for a region is defined as the ratio between the length and width of the minimum bounding rectangle of the region (smallest rectangle).

#### 2.6.7. Ellipse Axes

Ellipse axes are the longest (*Ra*) and shortest (*Rb*) ellipse axes; they share the same orientation and the same aspect ratio as the region. Ellipse axes are computed using normalized geometric moments, which can be computed according to the following formula:

$$M_{ij}=\frac{1}{F}\sum {}_{(r,c)\in R}{\left(r_{o}-r\right)}^{i}{\left(c_{o}-c\right)}^{j}$$

where *F* is the area of the region, *r*_0_ is the value of the center of gravity of the region along the rows, *c*_0_ is the value of the center of gravity of the region along the columns, *r*, *c* is the sums of pixels along the rows and columns, respectively, and *i* and *j* represent numbers that can be used to identify the type of normalized geometric moment.

Consecutively, *Ra* and *Rb* are expressed as:

$$Ra=\frac{\sqrt{8(M_{20}+M_{02}+\sqrt{{\left(M_{20}-M_{02}\right)}^{2}+4M_{11}{}^{2}}}}{2}$$

$$Rb=\frac{\sqrt{8(M_{20}+M_{02}-\sqrt{{\left(M_{20}-M_{02}\right)}^{2}+4M_{11}{}^{2}}}}{2}$$

#### 2.6.8. Compactness

The compactness of a region is a numerical quantity representing the degree to which a given shape is compact. It is computed using the following equation:

$$Compactness=\frac{P^{2}}{4F\pi }$$

where *P* stands for the perimeter of the region, and *F* is its area.

For a perfect circle, compactness should be equal to 1, while if the region is elongated or has holes, compactness should be larger than 1. The higher the compactness value, the more elongated or perforated the shape.

## 3. Results

Quantitative data concerning the morphological shapes and colors of the MPs found in the fish organs are summarized in Table 1. A total of 93 thresholded images out of 125 acquired images were further analyzed.

The shape descriptors measured using the MotiConnect 1.5.9.10-build-171215 software and computed based on CCL output are summarized in Table 2.

## 4. Discussion

### 4.1. General Physical Characterization

Among the morphological types, fibers were the most dominant (56.8%), followed by particles (42.4%) and pellets (0.8%) (Table 1). The average length and width of all identified particles (determined via the use of MotiConnect 1.5.9.10-build-171215 software) were 0.644 mm and 0.343 mm, respectively, while the mean length of the fibers was 1.266 mm (Table 2). The identified plastic items were divided into five size ranges according to their length. The dominant size range for the entire set of MPs (125 items) was 1–5 mm (44%), then 0.1–0.5 mm (32%), and 0.5–1 mm (17%). Among the fibers, particles, and pellets, the dominant size ranges were 1–5 mm (52%), 0.1–0.5 (45%), and 0.1 (100%), respectively. Regarding the MPs used for algorithmic assessment, the dominant size range was 1–5 mm (44%), then 0.1–0.5 mm (36%), and 0.5–1 mm (17%). Among the fibers, particles, and pellets, the dominant size ranges were 1–5 mm (53%), 0.1–0.5 (53%), and 0.1 (100%), respectively.

### 4.2. Length and Width of Particles

**Figure 6:**
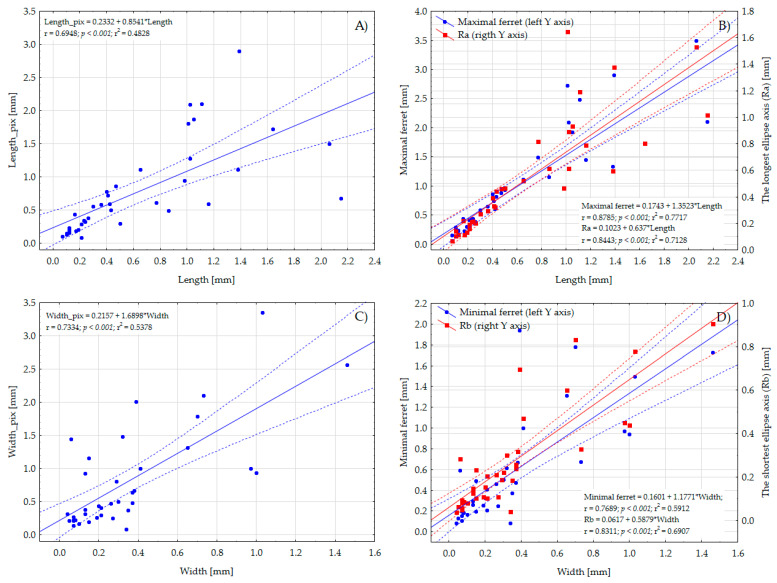
Linear regressions with confidence intervals expressing a directly proportional relationship between the length and width of particles (determined in MotiConnect 1.5.9.10-build-171215 software) and length (**A**), *Ra* (**B**), maximal ferret (**B**), width (**C**), *Rb* (**D**), and minimal ferret (**D**) (all determined algorithmically), respectively.

The average length and width of particles (determined via the use of the MotiConnect software) were 0.644 mm and 0.343 mm, respectively, while the average length and width of the MP items computed from binary images ranged between 0.513 mm (the longest ellipse axis) and 1.046 mm (maximal ferret) and between 0.264 mm (the shortest ellipse axis) and 0.796 (width of the smallest rectangle), respectively (Table 2). Although all algorithmic approaches compute linear measures that are significantly correlated with dimensions determined via manual assessment (*p* < 0.001, r in the range between 0.69 and 0.88, Figure 6), it seems that the best approximation of the length of the particle can be algorithmically achieved via the computation of the maximal ferret (r = 0.8785) or the longest ellipse axis (r = 0.8443), while the shortest ellipse axis, to the best extent, corresponds with the width of the MP items (r = 0.8311). As follows from Figure 6, through using ferret diameters or ellipse axes, the optimal mapping of linear measures of MP particles can be obtained since the majority of measured (width) and algorithmically computed values (ferrets, ellipse axes) are in the range of confidence interval of linear regression.

### 4.3. Area of Particles and Fibers

**Figure 7:**
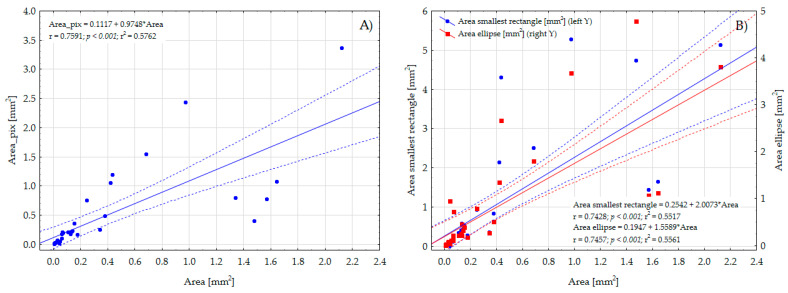
Linear regressions with confidence intervals expressing a directly proportional relationship between the area of particles calculated using the length and width of the MP items determined in MotiConnect 1.5.9.10-build-171215 software and the area calculated based on pixels (**A**), smallest rectangle (**B**), and the ellipse axes (**B**), respectively.

The average area of the plastic particles, determined using their length and width set manually in the MotiConnect 1.5.9.10-build-171215 software, was 0.355 mm^2^, while the average area of the particles (computed based on thresholded images) was 0.458 mm^2^, which is 29% higher. The average area of the particles computed algorithmically using the approach of the smallest rectangle or ellipse axes was 0.967 mm^2^ and 0.748 mm^2^, respectively (Table 2). For fibers, the average area computed using the pixels’ counting method was 0.058 mm^2^, while the MotiConnect software seems to be unsuitable for determining the area of fibers since it does not enable the precise computing of the width of the fiber along its length. As presented in Figure 7, the computed area (using the approach of the smallest rectangle or ellipse of the area analogous to an area of the region) of MP particles is significantly correlated (*p* < 0.001) with values derived from the MotiConnect software. According to the type of correlated areas, the Pearson correlation coefficient was around 0.75. The presented results indicate that MP pictures taken using any digital camera, after thresholding, can be successfully used to precisely determine the area of a variety of morphological shapes of plastic items (including shapes with slotted holes or even fibers), even when dedicated image processing software is not available or existing programs do not allow for the computation of such shape descriptors.

### 4.4. Fiber Length

**Figure 8:**
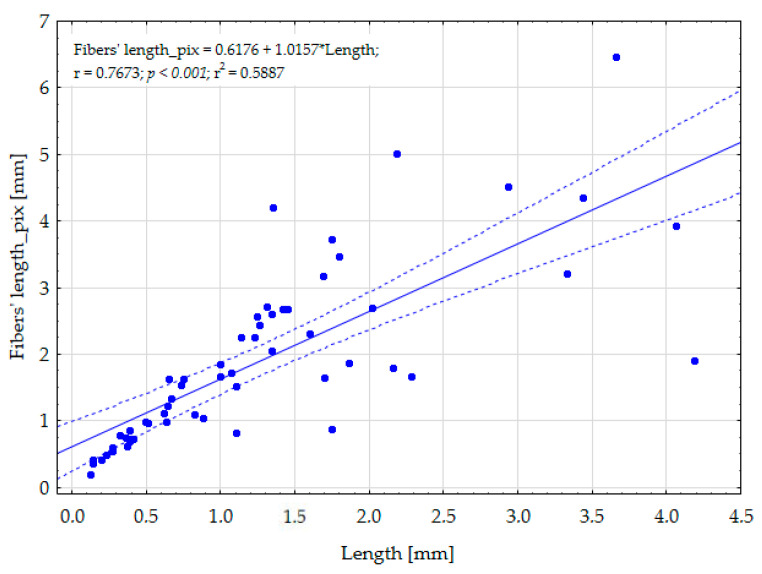
Linear regression with confidence interval expressing a directly proportional relationship between the length of fibers determined using MotiConnect 1.5.9.10-build-171215 software and the length of fibers computed using the length of the outer perimeter.

To approximate the length of the fiber, the outer perimeter of the region expressing the fiber’s shape was divided by two since the contour tracing algorithm starts from the given pixel and ends when the same pixel is reached again under certain conditions. The mean manually measured length of fibers was 1.266 mm. However, manually determining fiber length is problematic and highly time-consuming since it requires an operator to create the curve along the fiber. The average fiber length computed from the thresholded image was around 50% higher and equal to 1.903 mm. As presented in Figure 8, the length of the fibers computed using the value of the outer perimeter length fits satisfactorily (*p* < 0.001) to the length assessed by using the manually created curve method. Even if we take into consideration that the length computed from the thresholded image could somehow be overestimated due to the calculation approach, it is much faster and much more operator-independent. The overestimation is because the fiber itself is characterized by its width, while the outer perimeter calculation computes the output value as a distance around all outer contour pixels. When the outer perimeter length is simply divided by two, the overestimation caused by curvature at the end of the fiber could take place. Moreover, it needs to be emphasized that the presented approach could not be used to compute the length of the fibers that contain internal spirals. However, in our opinion, the advantages of computing fiber length from the thresholded image surpasses the disadvantages connected with the fiber length overestimation or spiral shapes.

### 4.5. Compactness and Circularity

**Figure 9:**
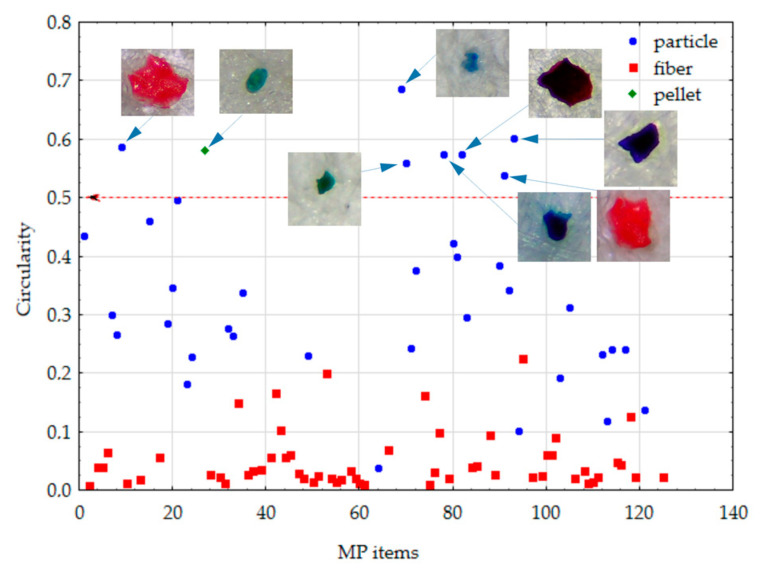
The circularity of MP items (the numbering shown on the *X*-axis corresponds to the numbering of images taken using the digital camera (125); after thresholding, images possessed their original numeration).

**Figure 10:**
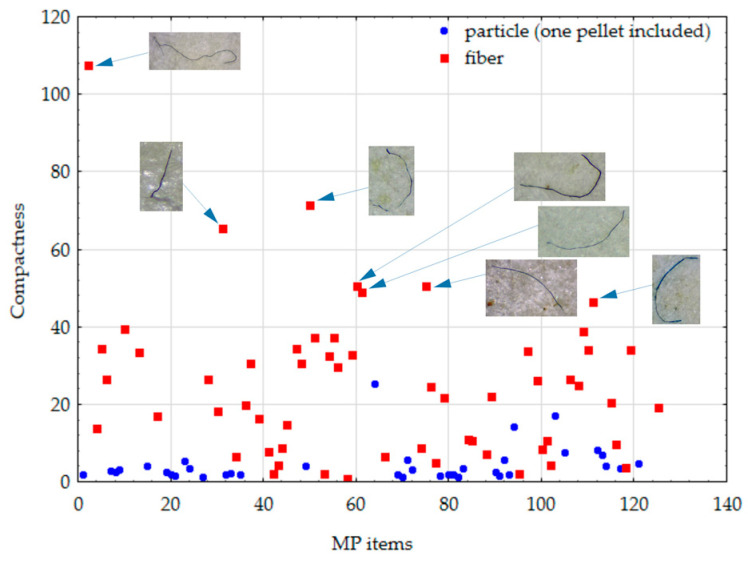
The compactness of MP items (the numbering shown on the *X*-axis corresponds to the numbering of images taken by the digital camera (125); after thresholding, images possessed their original numeration).

As mentioned above, compactness and circularity are shape descriptors that should be inversely correlated, measuring how closely the shape of the region approaches the shape of the circle and somehow quantifying elongatedness and the degree of perforation. The average circularity and compactness of particles computed from the thresholded image were 0.348 and 4.531, respectively, while the same set of shape descriptors for fibers consisted of a mean circularity equal to 0.051 and mean compactness equal to 24.931 (Table 2). Pearson’s correlation coefficient was used to quantify the relation between both shape descriptors for particles and fibers and generated values of −0.6162 (*p* < 0.001) and −0.6085 (*p* < 0.001), respectively. The U Mann–Whitney test (used for two independent group comparisons) confirmed the statistical difference in median circularity (U = 46.0, *p* < 0.001) and compactness (U = 205.0, *p* < 0.001) between the particles (median circularity = 0.31; median compactness = 2.97) and fibers (median circularity = 0.03; median compactness = 22.23). In Figure 9 and Figure 10, circularity and compactness computed for a set of 93 MPs shapes according to the particles and fibers are presented. Some additional images of MPs with maximal values of computed shape descriptors are also presented.

## 5. Conclusions

The results obtained in this study confirm that images of MPs taken by any digital camera in concert with a microscope can be successfully used to quantify shape descriptors of plastic items via a procedure consisting of digital image thresholding, binarization, the determination of connected components, and the computation of shape descriptors on a pixel level. Although no new image processing techniques have been proposed, the novelty of this study lies within the use of an open computer vision library and self-written C++ code to unify the determination of morphological features of MPs detected in various environmental or biological matrices, which, nowadays, is of high public interest. As a result, studies on MPs can be initiated worldwide in less equipped facilities.

The proposed approach can be used without the initial preparation of images that present items that are darker or lighter than the background; however, some additional steps, such as dilution, erosion, or the application of some histogram-derived thresholding methods, could further improve it. The best approximation of the length of the particle can be simply achieved by computing the maximal ferret (r = 0.8785) or the longest ellipse axis (r = 0.8443), while the shortest ellipse axis adequately correlates with the width of the MP items (r = 0.8311). An area of items computed via the use of the pixel counting method significantly (*p* < 0.001) correlates with the area computed via the multiplication of the approximated length and width of items; however, an approach that involves using thresholded image analysis enables one to also precisely compute the area of fibers or shapes possessing slotted holes. The problematic computation of fiber length can be overcome by estimating it using the item’s outer perimeter length. Circularity and compactness are inversely correlated shape descriptors that diversify particles and fibers. To the best of our knowledge, the proposed methodology has not been used to determine the morphological features of MPs as an alternative to more or less user-friendly commercial software. Moreover, as successfully shown, a wider range of useful shape characteristics, such as outer perimeter length, ferret diameters, or ellipse axes, can be used to qualify MPs of various shapes.

## Figures and Tables

**Table 1:** The number and percentage of MP items detected in fish organs according to their shape and color, as well as the number and percentage of MP images analyzed by the use of the CCL.

Color	MP [Items]						Total Detected	Total Analyzed
	Fiber		Particles		Pellet			
	Detected	Analyzed	Detected	Analyzed	Detected	Analyzed		
black	4	4	5	4	0	0	9 (7.2%)	8 (88.9%)
blue	54	49	19	14	0	0	73 (58.4%)	63 (86.3%)
green	4	0	7	7	1	1	12 (9.6%)	8 (66.7%)
pink	0	0	6	4	0	0	6 (4.8%)	4 (66.7%)
red	6	4	6	6	0	0	12 (9.6%)	10 (83.3%)
transparent	3	0	8	0	0	0	11 (8.8%)	0 (0%)
white	0	0	1	0	0	0	1 (0.8%)	0 (0%)
yellow	0	0	1	0	0	0	1 (0.8%)	0 (0%)
Total	71 (56.8%)	57 (80.2%)	53 (42.4%)	35 (66.0%)	1 (0.8%)	1 (100%)	125	93 (74.4%)

Note: The percentage of the total analyzed MP items was calculated with respect tothe total detected MP items.

**Table 2:** Basic statistics of the shape descriptors of the MP items found in the biological samples.

	Shape	N	Mean	Min	Max	S.D.
Length [mm]	particles	36	0.644	0.070	2.150	0.561
Width [mm]			0.343	0.040	1.460	0.330
Area [mm^2^]			0.355	0.005	2.122	0.558
Area_pix [mm^2^]			0.458	0.011	3.371	0.717
Area smallest rectangle [mm^2^]			0.967	0.015	5.288	1.508
Area ellipse [mm^2^]			0.748	0.012	4.787	1.167
Maximal ferret [mm]			1.046	0.153	3.492	0.864
Minimal ferret [mm]			0.564	0.082	1.941	0.505
Length_pix [mm]			0.784	0.085	2.902	0.690
Width_pix [mm]			0.796	0.082	3.350	0.760
*Ra* [mm]			0.513	0.072	1.647	0.423
*Rb* [mm]			0.264	0.039	0.908	0.233
Elongatedness			1.258	0.267	4.496	0.910
Outer contour [mm]			3.920	0.435	15.919	3.904
Circularity			0.348	0.039	0.685	0.161
Compactness			4.531	1.271	25.432	4.904
Length [mm]	fiber	57	1.266	0.120	4.190	1.005
Area_pix [mm^2^]			0.058	0.005	0.311	0.063
Maximal ferret [mm]			1.270	0.169	4.232	0.849
Minimal ferret [mm]			0.379	0.003	1.038	0.287
Length_pix [mm]			0.735	0.038	4.222	0.782
Width_pix [mm]			0.909	0.037	3.492	0.748
*Ra* [mm]			0.754	0.089	2.864	0.539
*Rb* [mm]			0.212	0.017	0.620	0.168
Elongatedness			1.924	0.059	15.421	2.880
Outer contour [mm]			3.806	0.397	12.950	2.662
Fibers’ length_pix [mm]			1.903	0.199	6.475	1.331
Circularity			0.051	0.009	0.225	0.049
Compactness			24.931	1.120	107.607	19.638

Note: S.D.—standard deviation; area_pix, area smallest rectangle, area ellipse maximal and minimal ferret, length and width_pix, *Ra* (longer axis of the ellipse), *Rb* (shorter axis of the ellipse), elongatedness, outer contour, circularity, and compactness are shape characteristics computed from CCL-based output.

## Data Availability

The data presented in this study will be available on request from the corresponding author after Paulina Piskuła Ph.D. thesis defense.
